# A Comparison between Chicago Classification Versions 3.0 and 4.0 and Their Impact on Manometric Diagnoses in Esophageal High-Resolution Manometry Cases

**DOI:** 10.3390/diagnostics14030263

**Published:** 2024-01-25

**Authors:** En Xian Sarah Low, Yen-Po Wang, Yong-Cheng Ye, Pei-Yi Liu, Kuan-Yi Sung, Hung-En Lin, Ching-Liang Lu

**Affiliations:** 1 Endoscopy Center for Diagnosis and Treatment, Department of Medicine, Taipei Veterans General Hospital, Taipei 11217, Taiwan; sarahlowex@gmail.com (E.X.S.L.); voxifera1225@hotmail.com (Y.-C.Y.); pyliu3456@gmail.com (P.-Y.L.); ioudanny520@hotmail.com (K.-Y.S.); scottenx@gmail.com (H.-E.L.); 2 Department of Medicine, Taipei Veterans General Hospital, Taipei 11217, Taiwan; 3 Department of Medicine, Ng Teng Fong General Hospital, National University Health System, Singapore 609606, Singapore; 4 Institute of Brain Science, National Yang-Ming University, Taipei 11221, Taiwan; 5 Faculty of Medicine, National Yang Ming Chiao Tung University School of Medicine, Taipei 11221, Taiwan; 6 Division of Gastroenterology, Department of Medicine, Fu Jen Catholic University Hospital, Taipei 24352, Taiwan; 7 Division of Gastroenterology, Department of Medicine, Taipei City Hospital Chongxing Branch, Taipei 10321, Taiwan

**Keywords:** high-resolution manometry, Chicago classification, ineffective esophageal motility, esophagogastric junction outflow obstruction, achalasia

## Abstract

High-resolution manometry (HRM) facilitates the detailed evaluation of esophageal motility. In December 2020, Chicago classification (CC) version 4.0 introduced modifications to improve consistency and accuracy. We conducted this study to compare the differences in the interpretations of HRM examinations between CC 3.0 and 4.0. Consecutive HRM records at a Taiwan tertiary medical center, including wet swallows and MRS performed in both supine and sitting positions from October 2019 to May 2021, were retrospectively reviewed and analyzed using both CC versions 3.0 and 4.0. A total of 105 patients were enrolled, and 102 patients completed the exam, while three could not tolerate HRM sitting up. Refractory gastroesophageal reflux disease (GERD) symptoms (*n* = 65, 63.7%) and dysphagia (*n* = 37, 36.3%) were the main indications. A total of 18 patients (17.6%) were reclassified to new diagnoses using CC 4.0. Of the 11 patients initially diagnosed with absent contractility, 3 (27.3%) were reclassified as having Type 1 achalasia. Of the 18 patients initially diagnosed with IEM, 6 (33.3%) were reclassified as normal. The incidence of diagnosis changes was similar in both the dysphagia and refractory GERD symptoms groups (21.6% versus 15.3%, *p* = 0.43). The use of CC 4.0 led to changes in the diagnoses of esophageal motility disease, irrespective of examination indications. Early adoption improves the accuracy of diagnoses and affects patient management.

## 1. Introduction

Esophageal high-resolution manometry (HRM) is frequently used for the evaluation of esophageal motility and pathology in clinical practice. Common indications include the evaluation of dysphagia, especially for the diagnosis of achalasia, preoperative workup before antireflux surgery, and monitoring treatment responses post-intervention [1]. Pressure sensors placed one centimeter apart along a catheter are inserted into the esophagus through the nose. This generates Clouse plots, which are esophageal pressure topography (EPT) charts, and facilitates detailed observations of peristalsis in the esophageal body, esophagogastric junction (EGJ) function, relaxation, and morphology [2]. HRM provides the Chicago classification (CC), a system that provides a standardized approach to interpreting HRM studies and facilitates categorizing of esophageal motility disorders to aid in management [3]. Patients were categorized into either having disorders with esophagogastric junction obstruction (EGJOO), major or minor disorders of peristalsis, or normal conditions. The first version of the CC system was introduced in 2009 after a systematic analysis of EPT patterns in 400 patients and 75 control subjects. Several key components of isolated swallows during the test are analyzed: the propagation and vigor of peristalsis, as well as the relaxation of the EGJ. This led to the first classification, which involved the objectification of achalasia and its three different subtypes, the identification of distal esophageal spasm, nutcracker esophagus, and EGJOO [4]. It underwent several revisions in 2012 [5], followed by version 3.0 in 2015 [6], with the most recent one being version 4.0, launched in December 2020 [7]. CC 4.0 (in 2021) introduced modifications to address several limitations that were noted in CC 3.0 after some years of clinical usage and experience. These were aimed at improving consistency, accuracy, and promoting increased standardization for effective clinical applications.

In CC 3.0, it was noted that ineffective esophageal motility (IEM) was frequently encountered (diagnosed when there were more than 50% ineffective swallows). However, there was a questionable correlation with symptoms experienced by patients. Hence, in CC 4.0, this was modified to require more than 70% ineffective swallows or at least 50% failed peristalsis with a normal integrated pressure (IRP) in order for a diagnosis of IEM to be made [8].

Chicago 4.0 has been significantly enhanced with a more thorough and comprehensive HRM protocol. Wet swallows are acquired in supine and upright positions, then followed by provocative tests to better differentiate esophagogastric junction outflow obstruction (EGJOO) from other disorders of peristalsis, such as hypercontractile esophagus, type III achalasia, or just normal peristalsis [9]. It is important to note that “a manometric diagnosis of EGJOO must always be considered clinically inconclusive” [1]. With this new classification, there are now more conditions to fulfil before this diagnosis is deemed relevant clinically. The role of complementary testing with a timed barium esophagram and/or functional lumen imaging probe (FLIP) was also discussed, together with the presence of reported associated symptoms by the patient. This update is important to reduce chances of overdiagnosis and unnecessary invasive treatment for patients labelled as having EGJOO based on CC 3.0.

The ramifications of the recent revision in the classification of esophageal motility disorders on both diagnosis and management are presently unclear. The heightened stringency of these diagnostic criteria raises questions regarding the direct clinical implications for manometric diagnoses within the same cohort of patients. Consequently, our research endeavors to scrutinize and quantify the influence of these more stringent criteria on the precision and applicability of manometric diagnose. We hope to shed light on the extent to which the altered classification system may affect clinical decision-making and therapeutic strategies in the realm of esophageal motility disorders.

## 2. Materials and Methods

From October 2019 to May 2021, records of patients who underwent esophageal HRM in a tertiary medical center were retrospectively reviewed. Our center had started to obtain HRM measurements in both the supine and sitting positions since October 2019.

Inclusion criteria of the study were adult patients aged above 18 and patients who managed to complete the entire HRM examination. Patients who received endoscopic (POEM/pneumatic dilatation) or surgical treatment, such as laparoscopic Heller myotomy (LHM), for esophageal diseases within 2 years before the HRM examination were excluded. While the effects of POEM or surgical treatment certainly last for longer than this arbitrarily defined period, this interval was set because it defines the time for different treatment episodes with new symptoms after the initial treatment and follow-up. We manually reviewed and analyzed the records to obtain manometric parameters and reviewed the diagnoses reached by both CC v 3.0 and 4.0. Patients’ demographic data, clinical symptoms, and indications for HRM were collected. Patients’ Eckardt score [10] and reflux symptom index (RSI) [11] were also collated, together with their responses to the Chinese GERDQ questionnaire (Chinese GERD-Q) [12]. Physicians would ask about patients’ symptoms and administer these questionnaires in Chinese.

Esophageal HRM was performed using a 32-circumferential pressure channels with a 16-impedance channels solid state catheter (Laborie, Medical Measurement Systems, Enschede, Netherlands). Patients were instructed to fast for 8 h prior to the procedure. If the patient was clinically suspected to have achalasia, we would fast the patient for 12 h prior. Topical lidocaine jelly is applied to both nostrils via a cotton swab.

In the sitting position, the manometry catheter is introduced through the nostril into the esophagus, and the HRM commences. The subsequent steps involve adjusting the catheter depth and then fixing its position to ensure optimal data acquisition precision. Following these preparatory measures, the patient is afforded a brief period of rest, allowing them to acclimate to the presence and sensation of the catheter. Then, a 30 s baseline resting pressure measurement is obtained in the supine position. This is followed by ten 5 mL wet swallows and then by three multiple rapid swallows (MRS). The patient is then asked to sit up and have their resting pressure measured first, as before, then ten 5 mL swallows are administered. This is followed by a rapid drink challenge (RDC). Resting pressure is then obtained for 1 min before the catheter is removed.

Statistical analyses were performed using SPSS version 19 (IBM SPSS Statistics for Windows, Version 19.0. Armonk, NY, USA). Parametric data are presented as mean ± SD unless otherwise stated. The analyses were performed using Student’s *t*-test or Chi-Square tests as appropriate, and statistical significance was defined as *p* < 0.05.

## 3. Results

In our study, involving 105 participants, 102 individuals successfully completed both the supine and sitting high-resolution manometry (HRM) tests. The gender distribution was balanced, with 56 males and 56 females, and the mean age was 53.88 years (with a standard deviation of 14.65 years). Notably, three participants experienced intolerance during the sitting HRM test and were unable to complete it.

A total of 56 patients (54.9%) were male, and the mean age was 53.88 years old (with a standard deviation of 14.65 years). The most common indication for HRM examination was refractory gastroesophageal reflux disease (GERD) symptoms, observed in 65 participants (63.7%). Dysphagia was the second most prevalent indication, reported in 37 participants (36.3%).

We then analyzed the patients based on these two main categories of symptoms: GERD and dysphagia (Table 1). Within the subset of patients presenting with dysphagia, a discernible pattern emerged, indicating a higher Eckardt score, a lower Chinese GERD-Q score, and comparable reflux symptom index (RSI) scores when juxtaposed with their counterparts exhibiting GERD symptoms.

An interesting dichotomy in procedural dynamics surfaced during the evaluation of these two symptomatology groups. Specifically, patients with dysphagia demonstrated a lengthier supine procedure time compared to those presenting with GERD symptoms. However, when taken as a whole (including both supine and sitting positions), the overall procedural times for both groups appeared convergent. It is noteworthy that, collectively, most of the procedures were efficiently accomplished within a notably brief timeframe of 30 min.

**Figure 1:**
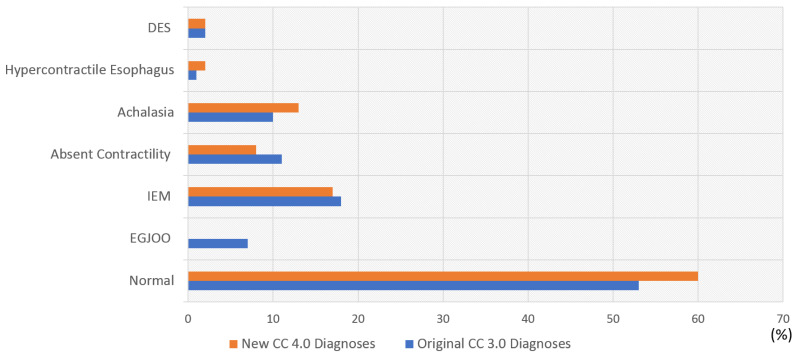
Distribution of manometric diagnoses using Chicago Classification version 3.0 and after Chicago classification version 4.0. DES: distal esophageal spasm, EGJ: esophagogastric junction, IEM: ineffective esophageal motility, EGOO: esophagogastric junction outflow obstruction, CC: Chicago classification.

**Figure 2:**
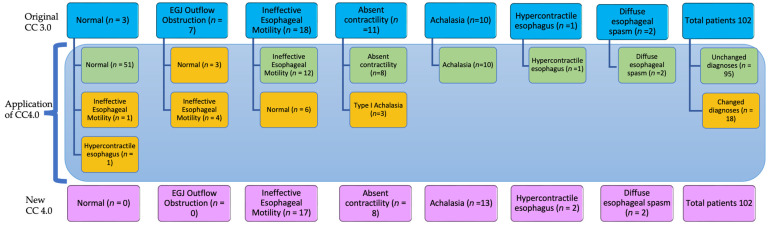
Before and after the application of CC 4.0: changes from the original CC 3.0 diagnoses: first row: blue boxes → original CC 3.0 diagnoses of patients. Middle rows: green boxes → unchanged diagnoses after application of CC 4.0, orange boxes → after application of CC 4.0. Last row: purple boxes → overall distribution of the new CC 4.0 diagnoses. EGJ: esophagogastric junction, CC: Chicago classification.

Most of the patients’ HRM diagnoses remained unchanged from CC 3.0 after application of CC 4.0. However, 18 patients (17.6%) were found to have a new manometric classification when CC 4.0 was applied (Table 2, Figure 1 and Figure 2).

### 3.1. Chicago Classification

#### 3.1.1. Esophagogastric Junction Outflow Obstruction

Within the subset of seven patients initially diagnosed with esophagogastric junction outflow obstruction (EGJOO), the application of CC 4.0 led to the revelation of new diagnoses (Figure 2). This included three patients classified as normal and four as having ineffective esophageal motility (IEM). The clinical course among these patients varied, with four experiencing improvement through conservative treatment, two were lost to follow-up, and one ultimately progressed to achalasia as symptoms exacerbated over time.

#### 3.1.2. Ineffective Esophageal Motility (IEM)

Amongst the 18 patients initially diagnosed with IEM with CC 3.0, 33.3% underwent a reclassification to normal with the application of CC4.0. Almost all of these patients had improvement of symptoms with conservative management.

#### 3.1.3. Absent Contractility

Amongst the initially classified cohort of 11 patients characterized as having absent contractility, a noteworthy 27.3% (3/11) underwent a significant reclassification, now falling under the category of Type 1 achalasia, subsequently prompting the recommendation of peroral endoscopic myotomy (POEM) (Figure 2). Notably, two of these patients underwent the suggested POEM procedure, and both experienced a tangible improvement in their symptoms, highlighting the clinical relevance of this reclassification.

#### 3.1.4. Normal

Two patients initially categorized as normal with CC 3.0 were reclassified with CC 4.0 and now diagnosed as having a hypercontractile esophagus and IEM, particularly following the application of the upright position protocol (Figure 2). Notably, both patients exhibited spontaneous resolution of symptoms during follow-up, underscoring the dynamic nature of esophageal motility disorders and the potential impact of positional considerations.

#### 3.1.5. Achalasia

It is essential to underscore that the diagnoses of all patients initially identified with achalasia under CC 3.0 remained unchanged upon transition to CC 4.0 (Figure 2), reaffirming the stability and robustness of achalasia diagnoses across these two Chicago classification iterations.

The incidence of a change in diagnoses was similar in both the dysphagia and refractory GERD symptoms groups (8/37 (21.6%) versus 10/65 (15.3%), *p* = 0.43). In the dysphagia group, patients diagnosed with normal HRM (1) IEM (2), absent contractility (3), and EGJOO (2) with CC 3.0 were reclassified with new diagnoses using CC 4.0. In the refractory GERD group, the patients who were reclassified originally had normal HRM (1), IEM (4), and EGJOO (5) diagnoses.

## 4. Discussion

In our study, an important observation emerged: the adoption of Chicago classification version 4.0 yielded changes in diagnoses of esophageal motility diseases, irrespective of the initial examination indications. This noteworthy finding underscores the substantive impact of transitioning from version 3.0 to version 4.0, highlighting the dynamic nature of diagnostic precision in the realm of esophageal motility disorders.

From our results, we found that the application of CC 4.0 had clinical implications on conditions that were previously diagnosed as EGJOO in the former classification. All seven patients with initial diagnoses of EGJOO were reclassified. This was a direct reflection of the more stringent criteria that CC 4.0 requires for EGJOO to be diagnosed. More conditions needed to be fulfilled—integrated relaxation pressure (IRP) had to be elevated in both the supine and upright positions in the presence of peristalsis, and supportive tests, such as barium swallow or FLIP, had to demonstrate obstruction at the EGJ, with clinical symptoms of chest pain and/or dysphagia. In our study, the original seven patients with EGJOO were reclassified as three normal studies and four with IEM.

There were no patients diagnosed with EGJOO with CC 4.0. This is in line with the relevant literature, where Visaggi et al. [13] demonstrated that, upon applying the CCv4.0 criteria, the prevalence of EGJOO decreased to 1.2%, suggesting that CCv4.0 offers improved specificity and accuracy in diagnosing EGJOO, leading to a more clear-cut identification of patients with this condition. Similarly, in the study by Alcalá-González et al. [14], 24% of patients with EGJOO were reclassified to a nonobstructive disorder, and the application of CC v 4.0 led to a change in diagnosis in 12% of the patients.

At first glance, what appears to stand out may be that the prevalence of IEM remained similar (Figure 1) after the application of CC 4.0, contrary to what the existing literature suggests, and seemingly going against what CC4.0 was meant to achieve with the new classification [8]. However, this is because four patients were reclassified from EGJOO to IEM, adding to the total tally of patients with IEM. In our study, six out of 18 (33%) patients with IEM (Figure 2) originally were reclassified to normal. This is in line with a similar study by Sallette et al. [15], where 24 out of 63 patients (38%) were reclassified as normal with CCv4.0. More recently, Carmel et al. [16] studied 152 patients who had manometric diagnoses of IEM with CCv3.0 and found that 39 (25%) were reclassified to normal; these patients with a change in diagnoses had fewer ineffective swallows and lower acid exposure time. It was concluded that fewer patients are diagnosed with IEM with CCv4.0.

The subsequent sections offer a closer examination of specific clinical cases, providing a detailed exploration of the details of their respective HRM examinations and illustrating the broader transformative impact of the updated classification system on individual patient outcomes.

### 4.1. Clinical Example: Originally EGJOO to Normal

Described below are the clinical details of a case whose initial diagnosis of EGJOO was overturned with CC 4.0.

**Figure 3:**
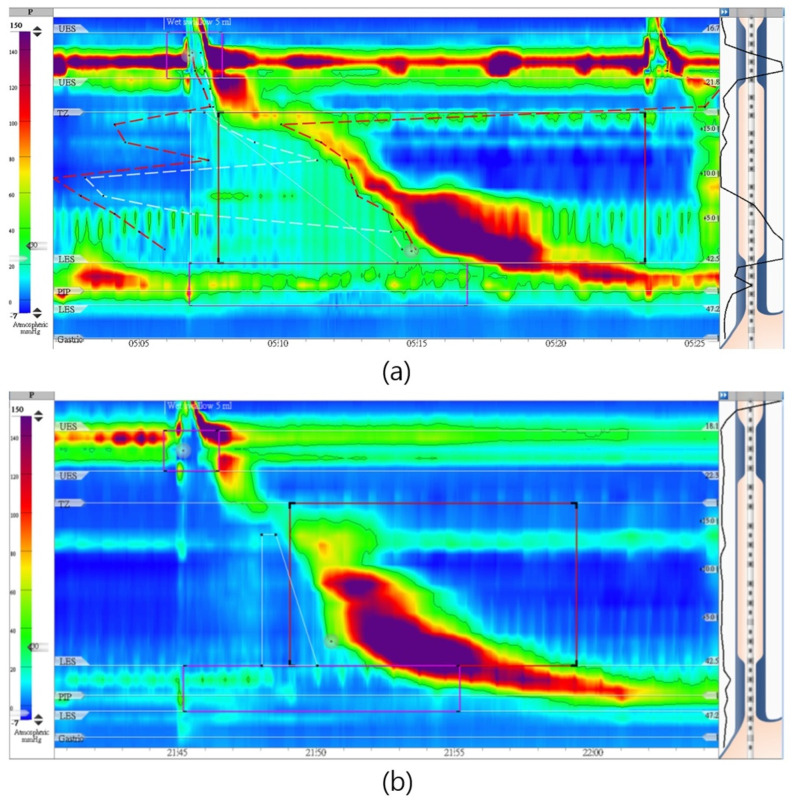
(**a**) A 66−year−old female with reflux and chest pain, having lower esophageal sphincter 4 sec integrated relaxation pressure = 32 mm Hg in supine position (Chicago 3.0) and (**b**) normalized to11 mm Hg in sitting position (Chicago 4.0).

We present the case of a 66-year-old female who, over the course of one year, experienced recurring episodes of chest pain associated with reflux. Initially diagnosed with EGJOO under CC 3.0, her symptoms were characterized by a high RSI score of 21 and a Chinese GERD-Q score of 27. In the supine position, her IRP-4 was notably elevated, at 32 mm Hg, as illustrated in Figure 3a. However, when the patient was repositioned to a sitting posture, her IRP-4 markedly decreased to 11 mm Hg, falling within the normal range. This observed positional discrepancy in IRP-4 values prompted a reconsideration of her diagnosis, and reevaluation under CC 4.0 resulted in a revised diagnosis, now categorized as normal. This case vividly underscores the clinical relevance of positional changes in diagnostic evaluations, especially in the context of possible EGJOO.

Clinically, this demonstrates that stricter criteria can indeed sieve out most patients (6/7, 85.7%) who would probably not have needed further specific intervention. For the patient, it means avoiding unnecessary interventions and associated procedures, contributing to a more streamlined and patient-centric healthcare experience. From the clinician’s perspective, this leads to a more efficient means of resource allocation. By identifying cases that genuinely warrant further specific intervention, clinicians can focus their efforts and resources on those patients, optimizing the utilization of healthcare resources. This not only enhances the efficiency of the diagnostic process but also has the potential to reduce the burden on healthcare systems, allowing clinicians to allocate their expertise where it is most needed.

However, the presence of one patient in our study who was eventually diagnosed with achalasia later shows that no test/classification is perfect.

Nevertheless, it is crucial to acknowledge the inherent imperfections in any diagnostic test or classification system, as exemplified by a specific case within our study: the presence of a singular patient, who was not initially marked as having achalasia in either CC 3.0 or CC 4.0. However, over the course of subsequent clinical follow-ups, a deterioration in symptoms became evident. A repeat HRM conducted one year later unequivocally diagnosed this patient with achalasia.

The evolving clinical trajectory of this case highlights the importance of longitudinal assessments, especially when confronted with ambiguous or inconclusive initial results. It prompts reflection on the dynamic nature of certain medical conditions, where symptom progression may unveil underlying pathologies that were not initially apparent. One pivotal learning point that emerged is the importance of reinforcing the practice of patients promptly reporting any worsening or new symptoms, even in cases where the initial tests yielded results deemed as “all clear.” Considering this, it might be prudent to consider a more proactive approach to follow-up care. Reminding patients to stay vigilant for any alterations in their symptoms and promptly seeking medical attention in such instances can be a pivotal aspect of patient education. This is especially relevant for cases where HRM findings are not entirely straightforward.

### 4.2. Clinical Example: Originally Absent Contractility to Achalasia

Additionally, three patients originally diagnosed with absent contractility were reclassified as having achalasia, which held significant clinical implications. Two of these patients underwent peroral endoscopic myotomy (POEM) and experienced marked improvement in symptoms. The third patient, with a history of achalasia and a laparoscopic Heller myotomy 20 years ago, missed the follow-up after being offered POEM in the clinic, leaving uncertainty about the course of her symptoms. The reclassification is crucial, as it could have led to potential delays in appropriate treatment, risking a less favorable response at a later stage. Described below is a description of one of the two patients where early intervention probably made a difference.

**Figure 4:**
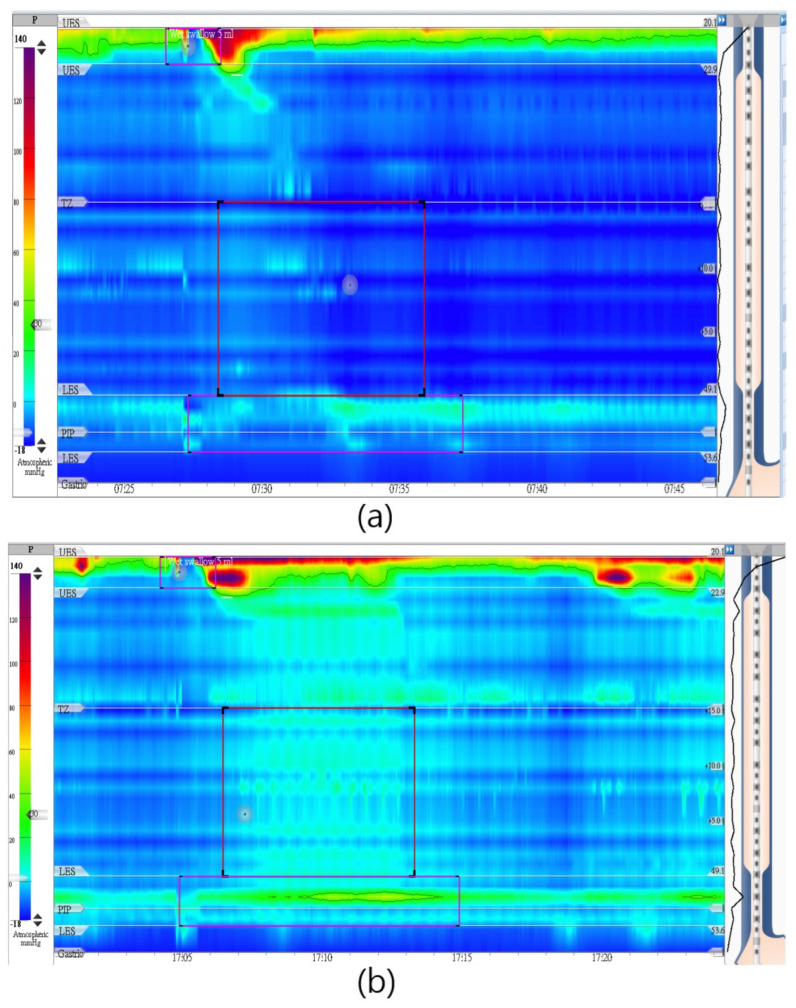
(**a**) A 29−year−old male patient who presented with dysphagia had normal lower esophageal sphincter 4 sec integrated relaxation pressure = 15 mmHg in supine position (Chicago 3.0) and (**b**) increased to 36mmHg in sitting position (Chicago 4.0).

A 29-year-old male patient, presenting with dysphagia symptoms persisting for 6 months, along with a history of longstanding reflux episodes, initially received a diagnosis of absent contractility under CC 3.0. His Eckardt score was 8, and his Timed Barium Esophagogram (TBE) was abnormal, producing a barium height of 110 mm in 5 min. His initial IRP-4 was 15mm Hg in the supine position (Figure 4a), with no peristalsis noted. After repositioning to the sitting position, his IRP-4 increased to 36 mm Hg (Figure 4b). With CC v 4.0, he now has a diagnosis of Type 1 achalasia. He underwent POEM with a good response.

### 4.3. Clinical Example: Originally Normal to Hypercontractile Esophagus

**Figure 5:**
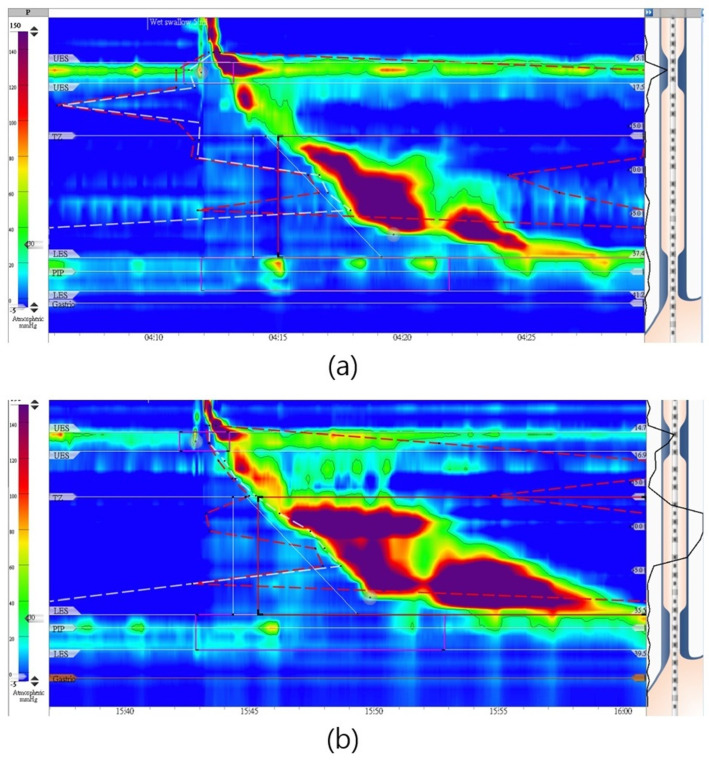
(**a**) An 81−year−old female who presented with dysphagia and heartburn had distal contractile integral: 5201 mmHg.sec.cm in supine position and (**b**) increased to 11,017 mmHg.sec.cm in sitting position.

We also want to highlight an interesting case featuring an elderly woman whose diagnosis changed following the application of Chicago classification version 4.0 (CC 4.0). This 81-year-old female had been grappling with symptoms of heartburn and mild dysphagia for over two decades and initially received a normal diagnosis under CC 3.0. Her Eckardt score was 4. In the supine position, her Distal Contractile Integral (DCI) was 5201 mm Hg s cm. (Figure 5a). After repositioning to the sitting position, her DCI increased to 11,017 mm Hg s cm (Figure 5b). With CC 4.0, this is now a diagnosis of hypercontractile esophagus. On clinical follow-up, she was found to have a significant esophageal diverticulum. It is not certain if the endoscopic finding was responsible for the difference in her manometric findings from a supine to a sitting position. Given the patient’s advanced age, a conservative approach was adopted in her management, refraining from specific interventions. Eventually, despite the absence of targeted interventions, her symptoms exhibited improvement over the course of the follow-ups.

Several limitations are inherent in our study. Firstly, the small sample size, limited to a single center and mainly focused on dysphagia patients, was partly influenced by the height of the COVID-19 pandemic. This is despite our center utilizing both supine and sitting positions during HRM prior to the publication of CC 4.0. Furthermore, the reimbursement guidelines for high-resolution manometry indications in Taiwan’s National Health Insurance are stringent. Patients must exhibit dysphagia suspected of an esophageal motility disorder following an OGD evaluation or have refractory gastroesophageal reflux disease (GERD) symptoms after medical treatment, specialist evaluation, and esophagogastroduodenoscopy assessment. Additionally, our study design is retrospective and observational; however, we prospectively obtained the patients’ demographic data, clinical profile questionnaire survey, and manometric examination data. Consequently, this approach limited the risk of selection bias and reporting bias. It is imperative to acknowledge that these limitations introduce inherent biases and limitations, and the observed trends should be interpreted within this context. Furthermore, the singular center focus may impact the generalizability of our findings to broader patient populations. The presence of the COVID-19 pandemic likely influenced patient recruitment and overall study dynamics, potentially introducing variables that could affect the external validity of our results.

To address these limitations and further elucidate the impact of CC 4.0, a prospective randomized study would be ideal. However, the feasibility of such a study, given practical and ethical considerations, is a significant challenge. While our study provides valuable insights, the outlined limitations underscore the need for cautious interpretation and emphasize the potential avenues for future research to enhance the robustness of our understanding in this complex clinical landscape.

## 5. Conclusions

In conclusion, our study underscores the significant impact of transitioning from Chicago classification version 3.0 to version 4.0 in the diagnoses of esophageal motility diseases, regardless of the initial examination indications. In our study, there was a reduction in the diagnosis of EGJOO and IEM, potentially avoiding overdiagnosis, unnecessary anxiety testing, and extending follow-up. Conversely, it was able to increase the pick-up rates of achalasia and tangibly affected patient outcomes. We thus believe that the application of CC 4.0 is pivotal, and early adoption is important due to its potential to influence clinical management.

## Figures and Tables

**Table 1:** Baseline characteristics.

Characteristics	All Participants (*n* = 102) Mean (S.D.) or *n*, %	Patients with Dysphagia (*n* = 37) Mean (S.D.) or *n*, %	Patients with Refractory GERD Symptoms (*n* = 65) Mean (S.D.) or *n*, % *n* = 65	*p* Value (Dysphagia vs. GERD)
Male	56, 54.9%	18, 48.9%	38, 58.4%	0.338
Age (years old)	53.88 (14.65)	55.46 (17.05)	52.98 (13.15)	0.415
Body height (cm)	163.94 (9.12)	162.38 (9.62)	164.83 (8.77)	0.193
Body weight (kg)	61.38 (11.22)	58.57 (10.02)	62.98 (11.62)	0.056
BMI (kg/m^2^)	22.78 (3.43)	22.16 (2.97)	23.13 (3.64)	0.172
Smoking	17, 16.7%	4, 10.8%	16, 24.6%	0.091
Alcohol use	20, 19.6%	3, 8.1%	14, 21.5%	0.080
Eckardt score	3.61 (2.55)	4.86 (3.28)	2.89 (1.67)	<0.001 *
Chinese GERDQ score	17.93 (7.72)	14.16 (5.87)	20.08 (7.86)	<0.001 *
RSI score	12.37 (8.03)	11.81 (8.1)	12.69 (8.02)	0.596
Procedure time (supine)	14 min 22 s	15 min 23 s	14 min 22 s	0.030 #
	(3 min 35 s)	(4 min 3 s)	(3 min 35 s)	
Procedure time (supine + sitting)	27 min 00 s	28 min 15 s	26 min 17 s	0.069
	(5 min 13 s)	(6 min 44 s)	(4 min 1 s)	

BMI: body mass index, GERDQ: gastroesophageal reflux disease questionnaire, GERD: gastroesophageal reflux disease, S.D.: standard deviation, RSI: reflux symptom index; * *p* < 0.001 between dysphagia and GERD patients; # *p* < 0.05 between dysphagia and GERD patients.

**Table 2:** Details of the 18 patients whose manometric diagnosis were reclassified after application of Chicago classification version 4.0.

No	Age	Gender	Diagnosis (CC 3.0)	Diagnosis (CC 4.0)	Indication	Management/Comments
1	71	F	Absent contractility	Type 1 achalasia	Dysphagia	Known achalasia, had LHM in 1989, suggested for POEM, lost to follow-up
2	57	F	EGJOO	Normal	Dysphagia	Improved with conservative management
3	81	F	Normal	Hypercontractile esophagus	Dysphagia	Improved with conservative management (had an esophageal diverticulum)
4	80	M	IEM	Normal	GERD	Improved with conservative management
5	37	M	IEM	Normal	Dysphagia	Improved with conservative management
6	69	F	EGJOO	Normal	GERD	Eventually diagnosed with achalasia later
7	30	M	Normal	IEM	GERD	Improved with conservative management
8	66	F	EGJOO	Normal	GERD	Improved with conservative management
9	46	F	EGJOO	IEM	GERD	Improved with conservative management
10	56	F	Absent contractility	Type 1 achalasia	Dysphagia	Improved with POEM
11	83	F	IEM	Normal	Dysphagia	Improved with conservative management
12	60	F	EGJOO	IEM	GERD	Lost to follow-up
13	66	M	IEM	Normal	GERD	Improved with conservative management
14	29	M	IEM	Normal	GERD	Improved with conservative management
15	25	M	EGJOO	Normal	Dysphagia	Lost to follow-up
16	51	M	IEM	Normal	GERD	Improved with conservative management
17	46	F	EGJOO	IEM	GERD	Improved with conservative management
18	28	M	Absent contractility	Type 1 achalasia	Dysphagia	Improved with POEM

CC: Chicago classification, IEM: ineffective motility, EGJOO: esophagogastric junction outlet obstruction, POEM: peroral endoscopic myotomy, F: female, M: male, GERD: gastroesophageal reflux disease, LHM: laparoscopic Heller myotomy.

## Data Availability

The data presented in this study are available on request from the corresponding author. The data are not publicly available due to Institutional Review Board’s regulation.
